# The influence of dielectric permittivity of water on the shape of PtNPs synthesized in high-pressure high-temperature microwave reactor

**DOI:** 10.1038/s41598-021-84388-2

**Published:** 2021-03-01

**Authors:** Marek Wojnicki, Magdalena Luty-Błocho, Przemysław Kwolek, Marta Gajewska, Robert P. Socha, Zbigniew Pędzich, Edit Csapó, Volker Hessel

**Affiliations:** 1 Faculty of Non-Ferrous Metals, AGH University of Science and Technology, Mickiewicza Ave. 30, 30-059 Kraków, Poland; 2 Department of Materials Science, Faculty of Mechanical Engineering and Aeronautics, Rzeszow University of Technology, Aleja Powstańców Warszawy 12, 35-959 Rzeszow, Poland; 3 Academic Centre for Materials and Nanotechnology, AGH University of Science and Technology, al. A. Mickiewicza 30, 30-059 Kraków, Poland; 4 Institute of Catalysis and Surface Chemistry Polish Academy of Sciences, Niezapominajek 8, 30-239 Kraków, Poland; 5 Faculty of Materials Science and Ceramics, AGH University of Science and Technology, al. A. Mickiewicza 30, 30-059 Kraków, Poland; 6 MTA-SZTE Biomimetic Systems Research Group, University of Szeged, Dóm tér 8, 6720 Szeged, Hungary; 7 Department of Physical Chemistry and Materials Science, University of Szeged, Rerrich B. tér 1, 6720 Szeged, Hungary; 8 School of Chemical Engineering and Advanced Materials, The University of Adelaide, Adelaide, Australia

**Keywords:** Nanoscience and technology, Nanoscale materials, Nanoparticles

## Abstract

In this paper, a novel method for the synthesis of Pt nanoparticles (PtNPs) using a microwave autoclave reactor is proposed. For benchmarking, the obtained results are compared with the traditional, batch method. A novel process window is proposed, which is the application of high-temperature and high-pressure. The main finding is that this only brings advantage, when the ionic strength of the system is enough low. It is explained, that at high pressure and high temperature, water behaves like only a slightly polar solvent, approaching a subcritical state. This reduces the electrostatic stabilization of the particles. Moreover, a change in the Pt particle shape is observed under high pressure and temperature conditions, suggesting that additional physical–chemical processes are involved.

## Introduction

Nanoparticles attract attention, mainly due to the fact, that they exhibit different properties than bulk materials. These properties are not linearly correlated to the particles size and do not follow traditional physics, therefore, it is difficult to predict the final properties of nanoparticles without further tests.

One of the reasons why nanoparticles are so interesting is the fact, that the active surface area increases with the decrease of the particle size. In other words, it is possible to synthesize catalysts with a high activity using a relatively little amount of active metal^1^. This is especially important when rare and precious metals are used^2^. The latter is the case of platinum. This metal exhibits excellent catalytic properties^3–5^. It is resistance to all mineral and organic acids is outstanding^6^ with the exemption of exposure to boiling sulfuric acid (temperature above 320 °C) and aqua regia. Therefore, this metal is frequently used as a catalyst in the chemistry/fertilizer industrial processes such as *e.g.* ammonia and nitric acid synthesis^7,8^. The latest research shows that Pt nanoparticles can also be used in fuel cells^9^, as well as effective and stable catalyst for selective hydrogenation of 5-hydroxymethylfurfural^10^.

Platinum nanoparticles (PtNPs) can be obtained via a chemical or physical route. In practice, however, the first one is mostly applied due to the lower costs of equipment and easy implementation. PtNPs are usually synthesized by chemical reduction of Pt(IV)^11^ or Pt(II)^12^ salts with sodium borohydrides^13^, iron(II)^14^, ethanol^15,16^, N-heterocyclic thiones^17^, plants aqueous extractsor^18^, other reductants^19^, in the presence of sodium citrate^20^ or other stabilizing agent^20^. The common way is carrying out the synthesis in batch reactor. Besides and as a more modern approach, several papers reported efficient PtNPs synthesis in microreactors^21^. For example, the application of a microreactor equipped with a back pressure regulator enables to heat the aqueous reaction mixture above 105 °C. The use of solvents of even higher boiling point allows the application of even higher temperature. On this path, Chen et al.^22^ applied an ethylene glycol–water solution (volume ratio 3:1), for PtNPs synthesis at ~ 140 °C. The synthesis time was *c.a.* 1 h.

In many applications (*e.g.* for catalysis), PtNPs with a small size are obtained via chemical reduction using strong reductant like the aforementioned sodium borohydride. The reduction process is rapid, yet it has limits in the scope of application. Unfortunately NaBH_4_ is not applicable when the nanoparticles are produced for *e.g.* medical use, due to the nature of this strong reductant and its oxidized product. More friendly reductants like ascorbic acid, glucose, citric acid, etc. usually lead to production larger particles and the process of nucleation and growth is much longer than in the case of sodium borohydride. Therefore, it would be very desirable to develop a method, where small and stable PtNPs are obtained in a short time using a reductant, and preferably a “mild” one.

In this paper, we applied trisodium citrate (TSC) to stabilize and synthesize PtNPs, via a faster reduction compared to citric acid. Application of the latter is less popular mainly because this is a weak carboxylic acid, and its deprotonation requires some time. Moreover, sodium citrate is known and well described in the literature mainly for AuNPs using the so-called Turkevich method^23–25^. The size of obtained gold particles varies from 5 to 25 nm depending on the adopted procedure. Unfortunately, this method is time-consuming. It is required to boil the reacting solution for circa1h to obtain colloid with deep red color. Even more, using other reductants for the fabrication of PtNPs may take an even longer processing time.

Thus, the Turkevich method was applied for AuNPs synthesis in microreactor, where efficient heating of a small volume of the solution allowed for quick Au(III) ions reduction and obtaining the red colloidal gold within few minutes^26^. The use of a microreactor for the synthesis of precious metals offers many advantages, yet also implies complications, which need to overcome. The latter are is related to the nucleation, sedimentation and adsorption on the microchannel walls^27^. Such an autocatalytic particle growth is disadvantageous for at least two reasons. The first one is related to the effect of the metal deposit, which begins to act as a heterogenic catalyst for the chemical reaction, changing its kinetics^28,29^. The other one, in turn, is related to the physical blocking of the microreactor channels by deposited metal. This is quite troublesome because in order to unblock the channel it is necessary to use concentrated acids or their mixtures, *e.g.* aqua regia in the case of gold or platinum. These adverse effects can be eliminated by appropriate modification of the surface of the microreactor channels^30,31^.

Therefore, we decided to “speed up” the synthesis of PtNPs not using the chemical and chemical engineering (microreactor) opportunities, but rather by changing the physical state of the processing solution. We propose to apply a microwave heated high-pressure and high-temperature reactor.

The use of microwave radiation in inorganic chemical synthesis began in the 1970s^32^. Among its several advantages, a significant acceleration of the chemical reaction compared to "classic" techniques (the use of sand, oil baths, heating jackets, etc.) should be considered as the most important one. Besides, because microwave radiation usually does not heat the reaction vessel but only the solvent and reagents, it is easier to achieve an even temperature distribution in the reactor, avoiding the large temperature gradients observed when using "classical" techniques^32,33^. The effect of acceleration of a chemical reaction can be qualitatively explained using the Arrhenius equation, and thus the major advantage of microwaves is to facilitate reaching unhabitual high temperatures for organic chemistry in a way that these are well defined.

The use of the microwave radiation increases the frequency factor and thus the reaction rate constant^32^. Two effects would be responsible for this phenomenon: enthalpy, associated with the provision of energy supplied by microwaves in the form of the vibrational energy of reacting particles, and entropy, associated with the arrangement of molecules^34^.

There are two mechanisms responsible for heating the reaction mixture by microwave radiation. The first one relates to the presence of ions in the reaction mixture. They move under the influence of Coulomb interactions with an alternating electric field. Their movements are accompanied by collisions with other ions and kinetic energy is converted into thermal energy^35^. Generating heat in this way is very efficient^32^.The other mechanism is associated with the rotation of electric dipoles (friction and collisions, *e.g.* between water molecules) under the influence of an alternating electric field. This effect is observed when the rotational motion of water molecules is incompatible with the radiation phase, which is given for the microwave frequency range. The more polar the solvent, the more efficient the heating. It can be assumed that during the synthesis of nanoparticles, the heating of the reaction mixture is carried out by both mechanisms, with a dipole mechanism dominating at low temperature, and ionic one at high. That is because the dielectric constant (relative permittivity) of water depends on the temperature and varies from 78 for 298 K to 20 at 573 K^32^.

The even heating of the reaction mixture depends on the volume and geometry of the reaction vessel. Microwave radiation is reflected from its walls and standing waves are generated, therefore some areas will be heated more strongly, others less so. This effect can be particularly evident when heating small samples^32^.

Accordingly, in this work we performed a series of studies to confirm, that the application of a high temperature and high-pressure reactor is beneficial for nanoparticles synthesis. The expected outcome of this research is a guideline which process parameters would promote to synthesize small and stable platinum nanoparticles.

## Experimental

PtNPs were synthesized in the following way. First, metallic platinum was dissolved in aqua regia, to obtain the platinum (IV) hexachloride complex. To remove the excess of nitric acid, the obtained complex was distilled several times and dissolving again using hydrochloric acid. Finally, the dry salt was obtained, and dissolved in deionized water. Thanks to this, a stock solution with the concentration 0.0763 M was obtained^36^. All other solutions were obtained by subsequent dilution of this stock solution in deionized water.

As a reducing and stabilizing agent, tri-sodium citrate salt (purchased from POCH, P.A.) was used. A solution of tri-sodium citrate was obtained, by the dissolution of this salt in deionized water (> 18 MΩ, Polwater).

The experiments were performed using two types of reactors. The first one termed batch reactor is in the fact the Simax glass bottle. The reagents were mixed in the bottle, magnetically stirred and heated using water bath on the hot plate (IKA Werke, model RCT basic). As a second type of reactor, we applied a microwave digestion system (Ertec, Magnum II). This device allows to heat up solutions using microwaves (frequency 2.45 GHz) with the maximum output power 600 W. This reactor enables safe operation in the pressure range from 1 to 50 bar, the maximum temperature permitted by the manufacturer is 573 K. All process equipment being in the contact with reagents are made of polytetrafluoroethylene (PTFE). Thanks to the microwave heating, high temperature and high pressure can be obtained. The total volume of the reactor is 108 mL. In all experiments, the vessel was filled with 20 mL of sample.

Zeta potential, as well as size and size distribution of the obtained PtNPs were determined using DLS method (Malver Zetasizer Nano ZS with 630 nm laser). All the measurements were repeated five times.

The changes in UV–Vis spectra were monitored using a Shimadzu PC2501 PC spectrophotometer. Transmission electron microscopy (TEM) investigations were carried out on an FEI Tecnai TF20 X-TWIN (FEG) microscope working at an accelerating voltage of 200 kV. For this purpose, the PtNPs were transferred onto a carbon-coated copper TEM grids from water suspensions via drop-casting method.

The Fourier-transformed infrared (FT-IR) spectra were registered using Nicolet 380 spectrometer, using potassium bromide (KBr, spectroscopic grade) for sample preparation. For this purpose, colloidal suspensions of NPs were instilled onto KBr powder, next the powder was dried for 24 h at temperature of 80 °C. The KBr base pellets were formed using a hydraulic press.

X-ray photoelectron spectroscopy (XPS) analysis was performed using an ultrahigh vacuum system equipped with a hemispherical analyzer (SES R4000, Gammadata Scienta, Sweden). As a source of incident energy of 1256.6 eV, the Mg Kα was applied. The spectrometer was calibrated according to ISO 15,472:2001 standard. More details about resolution, calibration as well as data fitting and analysis were described in our previous paper^37^.

The specific surface area of the PtNPs was determined with the Brunauer–Emmett–Teller (BET) method (Quantachrome, model Nova 1200). Nitrogen gas was used as adsorbate, assuming that single nitrogen molecule can cover 0.16 nm^2^ of the substrate area. The weight of specimen was 0.0089 g. To obtain this, the five syntheses were performed. When they were finished, the solid particles were found on the bottom of the reactor. Thus, solutions were decanted off and the particles rinsed with deionized water. This procedure was repeated three times to remove tri-sodium citrate.

## Results and discussion

### Mechanism of PtNP formation in the batch reactor

**Figure 1:**
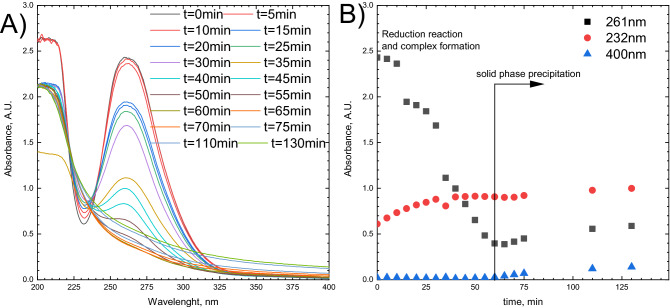
Kinetics of reduction of Pt(IV) chloride complex with TSC in the batch reactor: (**A**) UV–Vis spectra as a function of time; (**B**) kinetic curves. Experimental conditions: [Pt(IV)] = 1 × 10^−4^ M, [TSC] = 2 × 10^−3^ M, T ≈ 100 °C.

Thermodynamic calculation confirms that Pt(IV) chloride complex can be reduced with citric acid (H_3_Citric) and TSC according to the overall reaction (Eq. 1):

1 $$ 4.5PtCl_{6}^{2 - } + H_{3} Citric + 5H_{2} O\mathop{\longrightarrow}\limits^{{}}4.5Pt + 27Cl^{ - } + 6CO_{2} + 18H^{ + } $$

with equilibrium constants K_20°C_ = 3.6 × 10^257^ and K_300°C_ = 3.6 × 10^125^. Because at 20 °C this reaction occurs slowly, high temperature is required to obtain the metallic platinum in a reasonable time. The reduction in the batch reactor was conducted around 100 °C. Its progress was investigated spectrophotometrically (Fig. 1A,B).

The Pt(IV) chloride complex has two strong absorption bands in the UV spectral range, whereas TSC has not. Therefore, one of them, centered at 261 nm was used to investigate the reaction progress. The decrease of the absorbance with time can be directly converted to the changes of Pt(IV) chloride complex concentration using the Lamber-Beer law. The kinetic curve obtained for 261 nm has a sigmoidal shape (Fig. 1B). For the first 15 min, the change in the Pt(IV) chloride complex concentration is insignificant, but after 20 min, the reduction process accelerates. Increasing absorbance at 232 nm indicates that the reduction process consists of at least two steps. The intermediate, absorbing light at this wavelength is formed together with Pt(IV) chloride complex reduction. Its transformation to a solid phase is much slower. The solid phase appears in the solution, after circa 65 min. This was confirmed by increasing absorbance at 400 nm, corresponding to light absorption, and scattering by PtNPs. At the same time, the intermediate’s concentration remains constant. The increase of absorbance at 261 and 232 nm reflects the significant increase of background related to solid phase precipitation.

**Figure 2:**
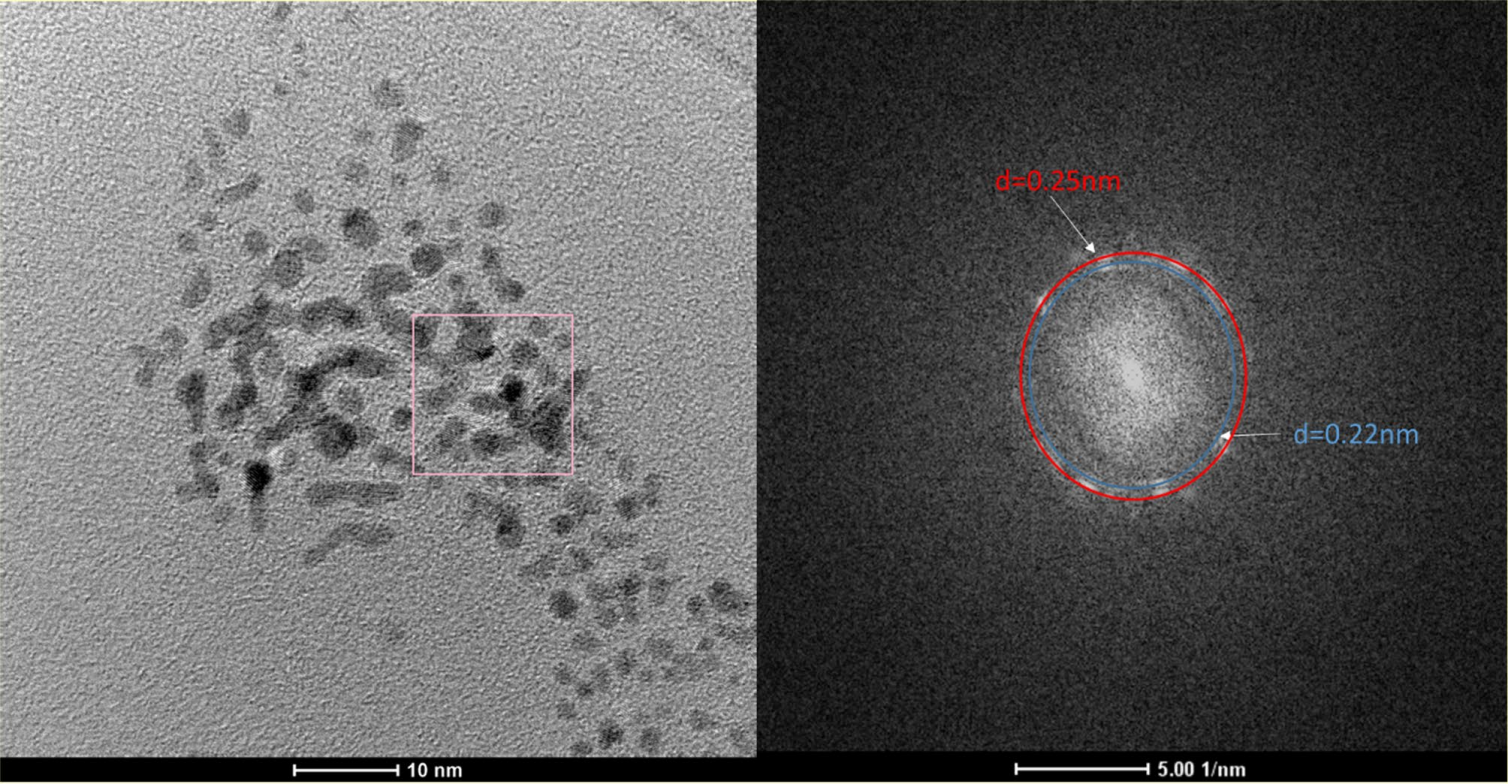
HR-TEM analysis of the PtNPs obtained in batch reactor. Conditions: [Pt(IV)] = 1 × 10^−4^ M, [TSC] = 1 × 10^−3^ M, synthesis time 60 min.

The obtained PtNPs were characterized with HR-TEM (Fig. 2). We clearly obtained that 80% of the particles have spherical shape. Therefore, further analysis of their size with DLS should give reliable results. FFT analysis of the micrographs revealed the presence of two phases. The first one, with interlayer spacing d_111_ = 0.22 nm was ascribed to the metallic Pt. The other one, with d = 0.25 nm could not correspond to PtNP. It might be ascribed to Pt_3_O_4_ (space group Pm-3n) but it requires further characterization of the reduction product. Nevertheless, microscopic analysis revealed formation of two solid phases in the system.

It can be concluded that the reduction Pt(IV) chloride complex with TSC in the batch reactor is rather slow and quite complex. It is of autocatalytic character, occurs via formation of an intermediate, and the reduction product is a mixture of two phases, where one of them is metallic platinum.

### Mechanism of PtNPs formation in the microwave autoclave reactor

#### The role of TSC in formation of PtNPs

**Figure 3:**
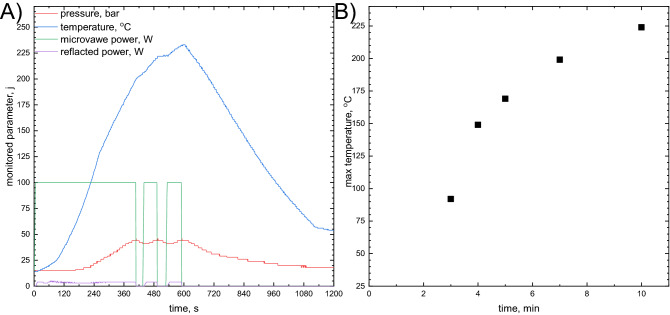
PtNPs synthesis in the microwave autoclave reactor: (**A**) parameters controlled during synthesis; (**B**) maximal value of temperature as a function of heating time; experimental conditions: [Pt(IV)] = 1 × 10^−4^ M, [TSC] = 2 × 10^−3^ M.

A microwave autoclave reactor was applied to increase the rate of Pt(IV) chloride complex reduction. Obviously, there is no possibility to investigate the concentration of the reagents during the reaction progress. The only measurable and controllable parameters are temperature, pressure, and a microwave power (Fig. 3A). In fact, temperature cannot be measured directly in the reaction mixture because of a Teflon wall between the sensor and the reaction mixture. Therefore, it is more convenient to use the pressure as the controlling factor. It can be measured more accurately and used for calibration of temperature when a substance with known temperature–pressure characteristics is heated. In our case, this calibration was done using deionized water.

The synthesis PtNPs was conducted with a pressure set between 45 and 50 bar. When being below the lower limit, the magnetron was switched on and the reaction mixture was heated with the maximal power. When the pressure reached 50 bar, the magnetron was switched off. This explains the rectangular shape of the microwave power and reflected power as well as nearly sinusoidal shape of pressure. Because of the thermal inertia of the reaction mixture and the Teflon-made reactor, the obtained temperature profile is smooth (Fig. 3A). The heating time, as defined as time necessary to achieve maximal value of temperature of the reaction mixture, was used as one of the varied parameters during synthesis (Fig. 3B).

Reaction mixtures with various concentrations of Pt(IV) chloride complex and TSC were prepared to establish the conditions, where PtNPs can be obtained using the microwave autoclave reactor. Further analysis was focused on the influence of TSC initial concentration, because it was from 10 to 200 times higher when compared to [Pt(IV)] and it behaves not only as the reductant, but also as an excellent agent stabilizing nanoparticles electrostatically. The electrostatic stabilization, however, is efficient only when the ionic strength of the solution is relatively low. Therefore, the influence of TSC on the size and stability of NPs is far from being trivial. On the one hand, the increase of TSC initial concentration should increase the reaction rate and decrease the NP’s size also improving their stability. On the other hand, the increase of TSC initial concentration significantly increases the ionic strength of the solution. This, in turn, causes coagulation and particle growth, especially at high temperatures.

**Figure 4:**
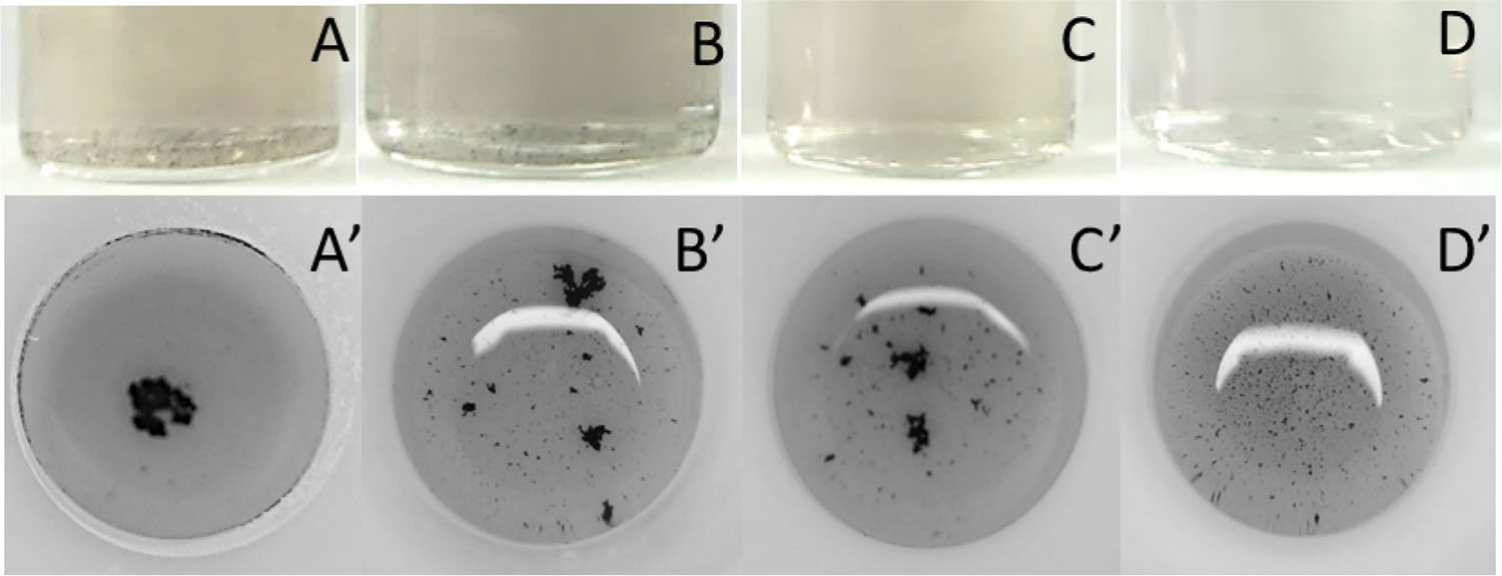
Photographical depiction of the reaction mixtures obtained after reduction in the batch (**A**–**D**), t = 60 min, T ≈ 100 °C and microwave autoclave reactor (**A′**–**D′**), t = 10 min, T_max_ ≈ 225 °C; chemical composition of the reaction mixture: (**A**, **A′**) [Pt(IV)] = 5 × 10^−4^ M, (**B**, **B′**) [Pt(IV)] = 2.5 × 10^−4^ M, (**C**, **C′**) [Pt(IV)] = 1 × 10^−4^ M, (**D**, **D′**) [Pt(IV)] = 5 × 10^−5^ M, [TSC] = 200 × [Pt(IV)] M.

Firstly, the reduction process was conducted in the batch and microwave autoclave reactor, when [Pt(IV)] = 5 × 10^−4^, 2.5 × 10^−4^, 1 × 10^−4^ and 5 × 10^−5^ M, with a 200-fold excess of the reductant when compared to the stoichiometric proportion [Eq. 1)]. As it was expected, stable colloids were obtained in the batch reactor (Fig. 4). When the reduction was conducted in the microwave autoclave, no stable colloids were obtained. Instead, large agglomerates of particles, probably platinum, were found at the bottom of the vessel. This means that the reaction mechanism in the microwave autoclaves is different when compared to the batch reactor and the 200-fold excess of TSC was much too high for the former.

**Figure 5:**
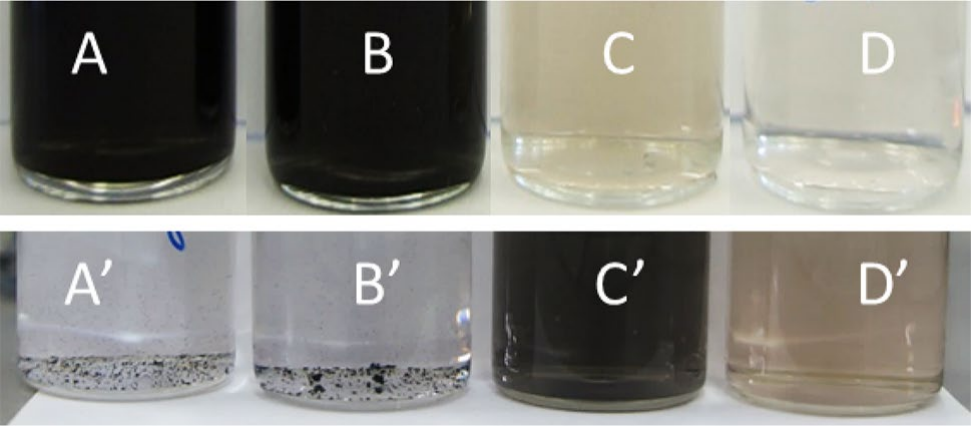
Photographical depiction of the reaction mixtures obtained after reduction in the batch (**A**–**D**), t = 60 min, T ≈ 100 °C and microwave autoclave reactor (**A′**–**D′**), t = 10 min, T_max_ ≈ 225 °C; chemical composition of the reaction mixture: (**A**, **A′**) [Pt(IV)] = 5 × 10^−4^ M, (**B**, **B′**) [Pt(IV)] = 2.5 × 10^−4^ M, (**C**, **C′**) [Pt(IV)] = 1 × 10^−4^ M, (**D**, **D′**) [Pt(IV)] = 5 × 10^−5^ M, [TSC] = 20 × [Pt(IV)] M.

Subsequently, a 20-fold excess of TSC was applied with the same initial concentrations of Pt(IV) chloride complex. The obtained results are shown in Fig. 5. When [TSC] was decreased, a black colloid was obtained at [Pt(IV)] = 5 × 10^−4^, 2.5 × 10^−4^ M in the batch reactor. The opposite effect was observed for the microwave autoclave reactor, where decreasing [TSC] enable obtaining stable colloids, when [Pt(IV)] = 1 × 10^−4^ and 5 × 10^−5^ M. Thus, the possibility of obtaining PtNPs in microwave autoclave reactor is demonstrated.

The size, distribution and Zeta potential of the PtNPs obtained using both reactors were compared for 20-fold excess of TSC (Table 1). High concentration of TSC increases the ionic strength of the solution, increasing PtNP’ size and decreasing their stability. The former was clearly observed for the synthesis in the batch reactor, whereas the latter not, because the uncertainties of Zeta potential were too high. Such a high uncertainties are probably obtained because the reduction product is non-homogeneous *i.e.* two Pt-containing phases were obtained (Fig. 2). Interestingly, the influence of ionic strength on the stability of PtNP, when the synthesis was performed in the microwave autoclave, was much stronger *i.e.* it was possible to obtain the stable colloid only at lower values of the ionic strength compared to batch reactor.

**Table 1:** Radius and Zeta potential of PtNP obtained at 20-fold excess of TCS.

[Pt(IV)], M	Ionic strength, M	Batch reactor		Microwave autoclave	
		r, nm	Zeta, mV	r, nm	Zeta, mV
5 × 10^−4^	0.046	6.9 ± 1.9	− 38 ± 9.3	*	*
2.5 × 10^−4^	0.023	5.0 ± 1.9	− 31.9 ± 6.5	*	*
1 × 10^−4^	0.009	2.0 ± 0.4	− 34.3 ± 7.0	4.1 ± 1.3	− 33.5 ± 2.0
5 × 10^−5^	0.005	2.0 ± 0.5	− 20.7 ± 11.7	1.1 ± 0.3	− 35.3 ± 1.7

*The colloid was not obtained.

**Figure 6:**
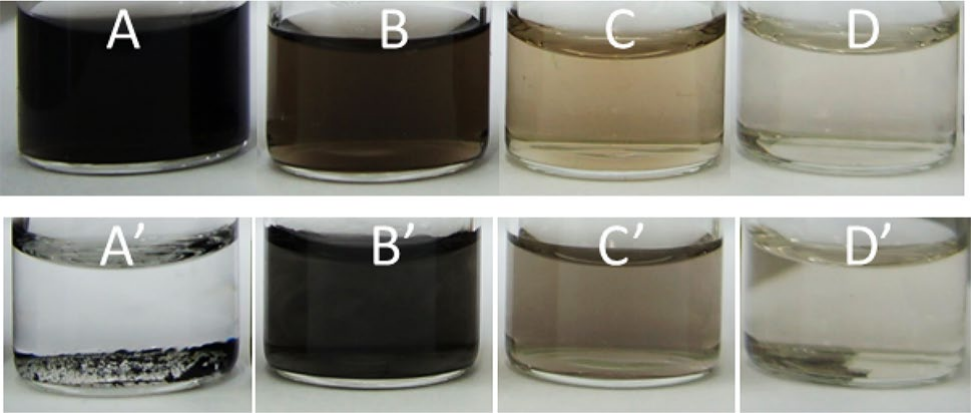
Photographical depiction of the reaction mixtures obtained after reduction in the batch (**A**–**D**), t = 120 min, T ≈ 100 °C and microwave autoclave reactor (**A′**–**D′**), t = 10 min, T_max_ ≈ 225 °C; chemical composition of the reaction mixture: (**A**, **A′)** [Pt(IV)] = 5 × 10^−4^ M, (**B**, **B′**) [Pt(IV)] = 2.5 × 10^−4^ M, (**C**, **C′**) [Pt(IV)] = 1 × 10^−4^ M, (**D**, **D′**) [Pt(IV)] = 5 × 10^−5^ M, [TSC] = 10 × [Pt(IV)] M.

The great influence of the ionic strength on the stability of PtNP produced in the microwave autoclave reactor was further confirmed when the tenfold excess of TSC was applied. The colloid was unstable only when [Pt(IV)] = 5 × 10^−4^ (Fig. 6, Table 2). Thus, small PtNPs with narrow size distribution can be obtained at relatively low values of ionic strength. When the concentration was equal to 0.012 M, rather big nanoparticles were obtained, at the expense of having a high value of uncertainty of their radius. This suggests that there is a mixture of big and small nanoparticles. This seems to be reasonable, and consequently when the ionic strength is > 0.012 M, only big particles were obtained, and the colloid was unstable. When the ionic strength was < 0.012 M, a stable colloid with small nanoparticles were obtained.

**Table 2:** Radius and Zeta potential of PtNPs obtained at tenfold excess of TCS.

[Pt(IV)], M	Ionic strenght, M	Batch reactor		Microwave autoclave	
		r, nm	Zeta, mV	r, nm	Zeta, mV
5 × 10^−4^	0.024	5.7 ± 1.5	− 34.4 ± 2.0	*	*
2.5 × 10^−4^	0.012	3.0 ± 0.7	− 36.3 ± 1.9	24.1 ± 12.9	− 50.6 ± 5.1
1 × 10^−4^	0.005	2.8 ± 0.5	− 35.8 ± 1.5	5.0 ± 1.3	− 34.6 ± 9.2
5 × 10^−5^	0.002	2.7 ± 0.6	− 33.9 ± 2.0	1.7 ± 1.3	− 22.4 ± 7.3

*The colloid was not obtained.

**Figure 7:**
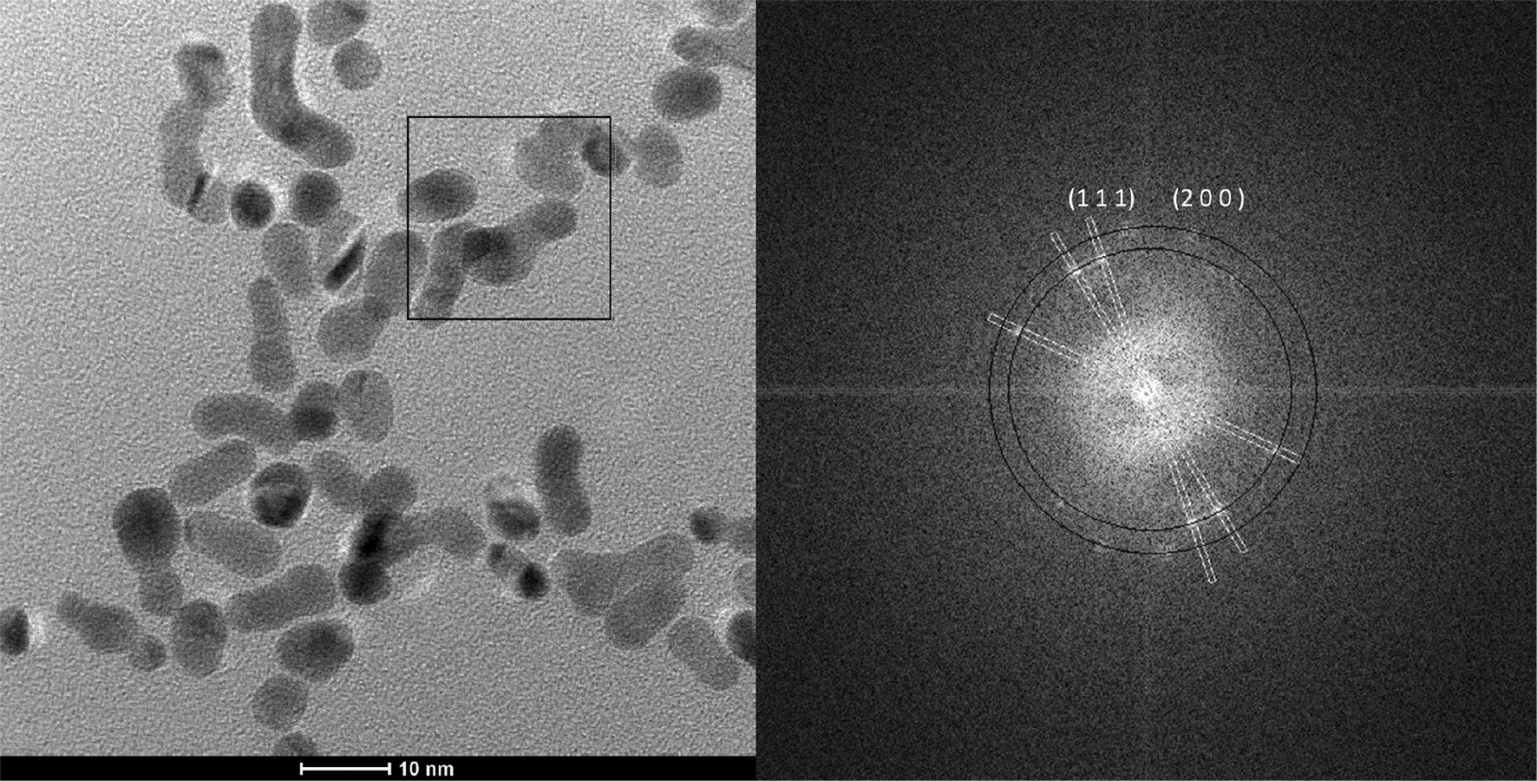
HR-TEM analysis of the PtNPs obtained in the microwave autoclave reactor at [Pt(IV)] = 1 × 10^−4^ M, [TSC] = 1 × 10^−3^ M, t = 10 min, T_max_ = 225 °C.

In fact, the DLS analyses do not confirm the phase composition of the obtained colloids. The formation of PtNPs using the microwave autoclave reactor was proved with HR-TEM. FFT analysis revealed two reflexes that were unambiguously assigned to Pt(200) and Pt(111) crystallographic planes. PtNPs are not spherical, but rather elongated, with their length around 2–3 times their diameter (Fig. 7). Controlling the shape of the nanoparticles in Pt(IV)-TSC system is possible. Also, it should be underlined, that the contrast intensity of all PtNPs is equal independently to the direction of analysis. It suggests that the nanoparticles are flat and thin. Gonzalez et al. obtained tetrahedral and octahedral PtNP, due to preferential adsorption of hydrated citrate anion on the Pt(111) surface. Three dehydrogenated carboxylic groups of citrate were bound to platinum in the bidentate configuration^38^.

PtNPs were synthesized at high temperature. Generally, such conditions enhance desorption rather than adsorption processes, provided that adsorbate-adsorbent interaction is of electrostatic nature, *i.e.* weak. Consequently, spherical PtNPs with the most densely packed, i.e. energetically favorable (111) plane on the surface should be obtained. The elongated shape of PtNPs with (200) and (111) planes exposed suggests the strong chemical bonding between citrate carboxylic groups and Pt atoms. This probably occurs at the (200) plane, because its surface energy is higher when compared (111), and stabilized the former plane. Attard et al. obtained similar result. They observed that at 100 °C formation of Pt(100) planes is strongly disfavored when compared to Pt(111)^39^.

**Figure 8:**
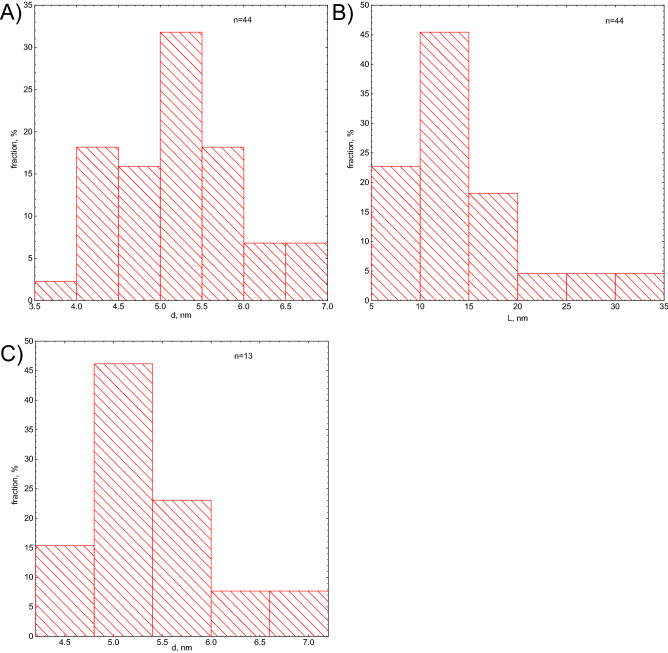
Size distribution determined from HR-TEM, (**A**, **B**) for elongated nanoparticles, (**C**) for spherical nanoparticles.

The HR-TEM images were used to determine the diameter and length of elongated nanoparticles (see Fig. 8A,B), where d corresponds to diameter and L to the length of the nanoparticles. The results are in good agreement with the DLS results (see supplementary materials Fig. 3SA, B, C). Figure 8C, corresponds to spherical nanoparticles.

**Figure 9:**
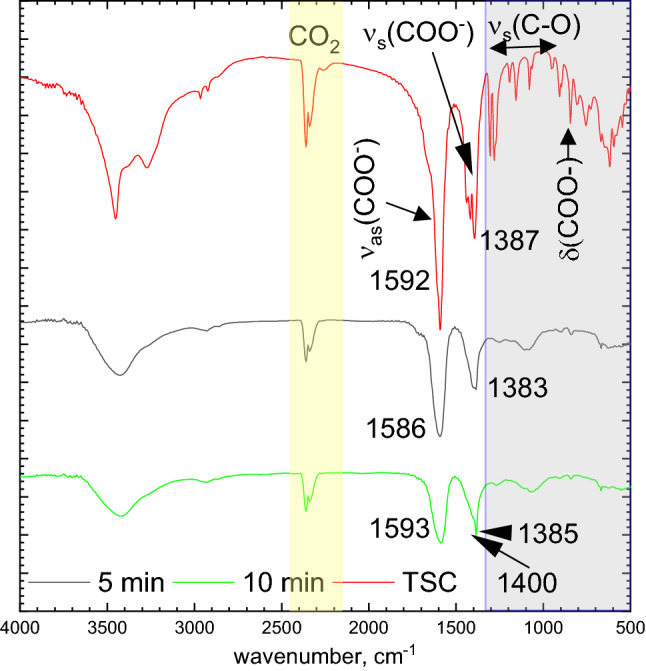
FT-IR spectra registered for the dried colloid obtained when [Pt(IV)] = 1 × 10^−4^ [TSC] = 2 × 10^−3^ M, T_max_ = 225 °C and TSC for comparison.

Adsorption of citrate anions on the PtNPs surface was qualitatively confirmed with FT-IR spectroscopy. Obtained results are shown in Fig. 9. Peaks ascribed for carboxylate asymmetric, v_as_ (COO^−^) and symmetric stretching v_s_ (COO^−^) appear at 1592 cm^−1^ and 1387 cm^−1^ respectively for pure TSC. The former was shifted only slightly, to 1592 cm^−1^ whereas the latter was split into two peaks at 1385 cm^−1^ and the second one at 1400 cm^−1^. Wulandari et al*.* characterized citrates adsorbed on gold and silver nanoparticles. They observed that this shift varied depending on the metal^40^. Thus, the observed changes in the v_as_(COO^−^) peak position can be ascribed to the adsorption of carboxylic groups of TSC on PtNPs.

#### The role of reduction temperature

**Figure 10:**
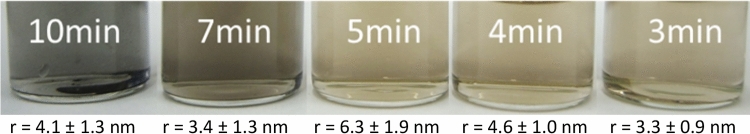
Photographical depiction of the colloids obtained after reduction in the microwave autoclave reactor, [Pt(IV)] = 1 × 10^−4^ [TSC] = 2 × 10^−3^ M.

Once the stable PtNPs were obtained in the microwave autoclave reactor it was interesting to study the influence of temperature *T*_max_ on their size. This temperature is related to heating time (Fig. 3B). Stable colloids, with the same NPs’ radii, were obtained regardless of temperature (Fig. 10), when [Pt(IV)] = 1 × 10^−4^ M and [TSC] = 2 × 10^−3^ M. Interestingly, they have different color. Because PtNPs do not exhibit surface plasmon resonance in visible range of the spectrum, the change in their color remains unclear.

**Figure 11:**
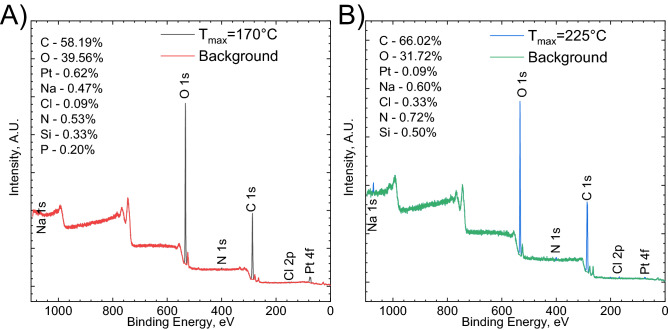
The survey spectra of two samples [Pt(IV)] = 1 × 10^−4^ [TSC] = 2 × 10^−3^ M: (**A**) T_max_ = 170 °C; (**B**) T_max_ = 225 °C.

This is explained with the information provided by the XPS measurement for the two colloids obtained at *T*_max_ = 170 and 225 °C (see Fig. 11A,B).

**Figure 12:**
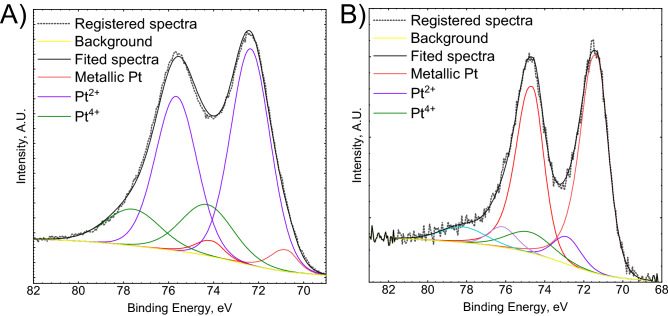
Pt 4f. photoelectron spectra registered for the colloids obtained when [Pt(IV)] = 1 × 10^−4^ [TSC] = 2 × 10^−3^ M: (**A**) T_max_ = 170; (**B**) T_max_ = 225 °C.

The analysis of a high-resolution Pt 4f_7/2_ spectrum brings information about the chemical structure of PtNPs (Fig. 12). When the colloid was obtained at *T*_max_ = 170 °C, dominating the oxidation state of platinum, 72.2 at.%, can be assigned to Pt^2+^ in the form of PtO or Pt(OH)_2_, with a binding energy BE = 72.6 ± 0.3 eV and well separated spin–orbit components (Fig. 12A). Much smaller amount of Pt^4+^, 22.6 at.%, in the form of PtO_2_ or Pt(OH)_4_, was also detected, BE = 74.5 ± 0.2 eV. There was only 5.3 at.% of metallic platinum, BE = 71.0 ± 0.3 eV^41^. When *T*_max_ = 225 °C, however, the specimen contained mostly platinum, 79.3 at.% with 9.3 at.% of Pt^2+^ and 11.5 at.% Pt^4+^ (Fig. 12B). XPS analysis revealed that at lower temperature the reduction product is mostly composed of Pt oxide/hydroxide in the form of nanoparticles, whereas at higher temperature, PtNPs were obtained with certain admixture of Pt oxides/hydroxides. This explains the different color of the colloids obtained at different temperature. The reduction mechanism is complex and probably involves hydrolysis of Pt(IV) chloride complex and subsequent reduction of hydrolyzed species to metallic platinum with formation of Pt(II) oxide/hydroxide as the intermediate. This mechanism could be also valid for the synthesis in the batch reactor, where certain an oxidized form of platinum was detected together with PtNPs.

**Figure 13:**
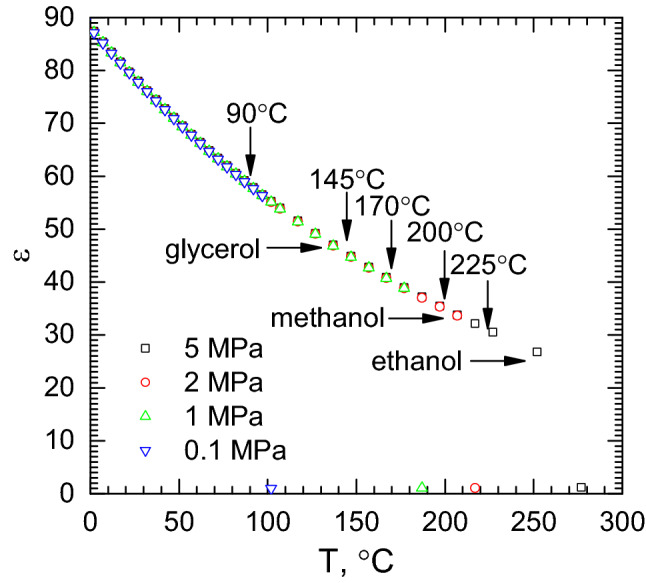
Dielectric permittivity of water as a function of temperature and pressure, data from^43^; vertical arrows indicate maximal temperatures during synthesis, horizontal ones show dielectric constant of selected organic solvents at room temperature^44^.

The influence of temperature on the reaction process is complex. Increasing temperature facilitates formation of PtNPs. At the same time, the reaction conditions change with the increasing temperature because the dielectric permittivity of water decreases. When the boiling point is achieved, the permittivity drops to *c.a.* 1. (Fig. 13). In other words, the higher temperature, the water behaves as a less polar solvent. This probably causes precipitation of TSC in the reactor during synthesis affecting the reduction kinetics. Obviously, this cannot be observed because TSC dissolves again during cooling. At the same time, ε virtually does not depend on pressure, in the range between 0.1 and 5 MPa. If TSC precipitates from the solution before the Pt(IV) chloride complex reduction, it may behave as nucleation centers for PtNPs affecting nanoparticles’ size. Usage of sparingly soluble salts as a shape controlling agent was demonstrated^42^. At the same time, the reductant concentration decreases with increasing temperature and again increases with decreasing temperature. Thus, more detailed study of the influence of heating parameters on the reaction mechanism should be conducted.

### Characterization of material deposited from unstable colloids

As it was shown above, the process of PtNPs synthesis in microwave autoclave reactor leads to formation of the black deposit, when the ionic strength is too high (Fig. 4). Interestingly, the volume of this deposit was unexpectedly high when compared to the relatively low initial concentrations of Pt(IV) chloride complex in the solution. This suggests a relatively high surface area of the deposit and its potential application *e.g.* as a catalyst. Analysis of BET were performed using a five-point approximation. The BET surface area was equal to 31.99 ± 1.59 m^2^/g which in fact is not very high value. It should be noted the density of Pt is equal to 21 g/cm^3^ compared to carbon in the form of graphite 2.2 g/cm^3^ for graphite. The nanoparticles are bulk, not porous. The determined surface area complies with the theoretical calculations. It corresponds to a 4.4 nm radius for PtNPs (see supplementary materials). Such nanoparticles can be easily obtained using presented method.

**Figure 14:**
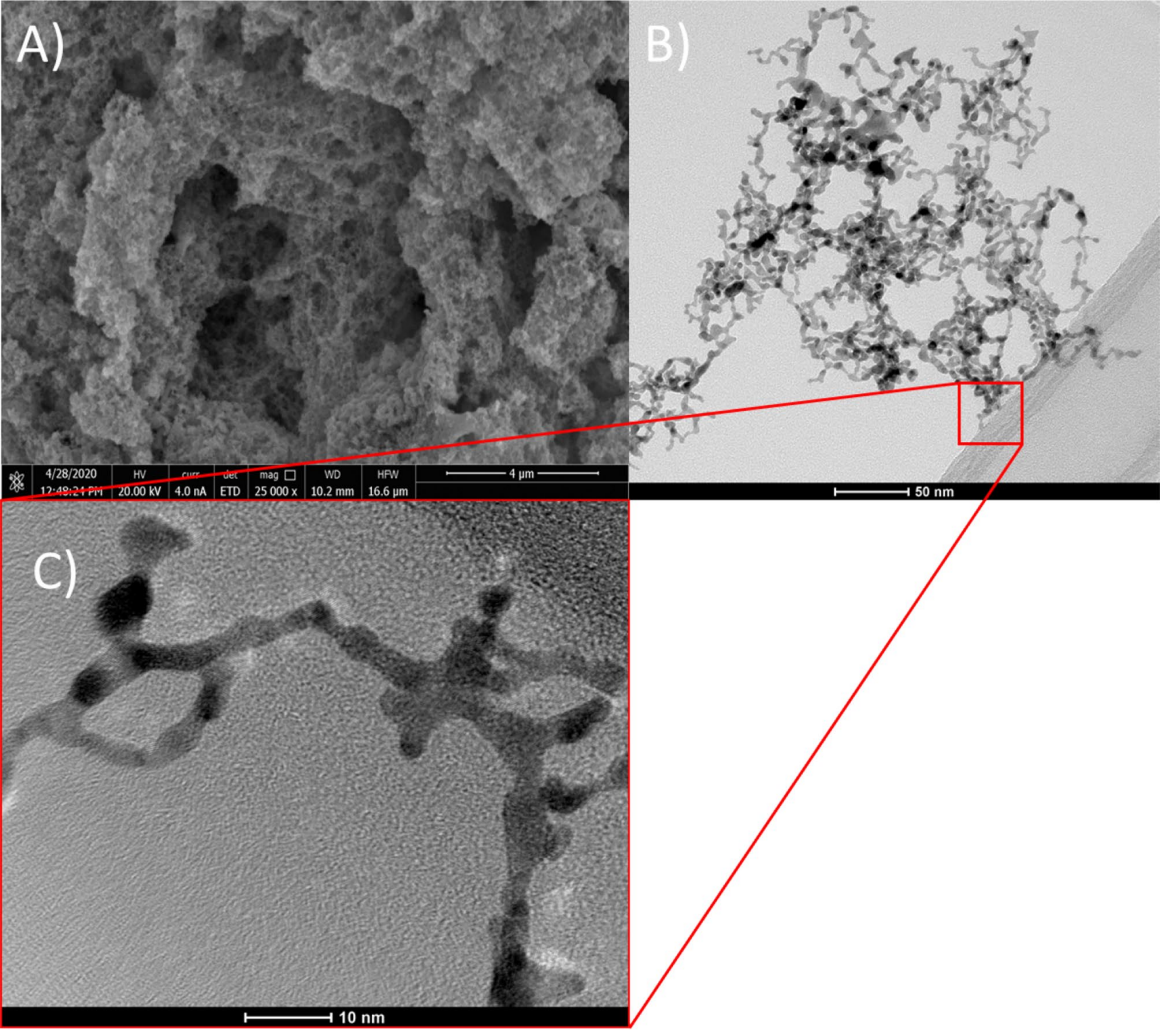
The sponge-like structure obtained when [Pt(IV)] = 5 × 10^−4^ M, [TSC] = 10 × [Pt(IV)] M, t = 10 min, T_max_ = 225 °C: (**A**) SEM micrograph; (**B**) and (**C**) HR-TEM micrograph.

SEM analysis suggest that the material is sponge-like, consisting of small crystals (see Fig. 14A). Interestingly, the sponge-like structure was easily dispersed using an ultrasonic bath. A stable colloid was formed and analyzed with HR-TEM (Fig. 14B,C). The nanoparticles are of elongated shape and seem to be rather thin because they were overexposed with the microscopic beam. These particles are light grey. It can be concluded that even at high ionic strength it is possible to obtain PtNPs, but the sponge-like deposit must be dispersed. In addition, these nanoparticles seem to be more elongated when compared to those obtained at lower ionic strength. This suggests that the TSC concentration, together with temperature, is an important parameter enabling controlling the shape of PtNPs.

## Conclusions

1. It was found, that using a microwave high-temperature high-pressure reactor the synthesis of PtNPs can be accelerated.
2. Thanks to the fact, that the reaction rate significantly depends on the temperature green reductant (usually weak) can be applied. Following this, PtNPs can be synthesized using biocompatible reductant, and may not require further purification process in case of application in medicine.
3. The research confirms that the synthesis of PtNPs in the presence of TSC is a multi-step process. In the first step, insoluble platinum compounds, probably hydroxides, precipitate. Then they are reduced to the metallic phase.
4. It is well, known that the hydroxides are thermodynamically unstable. In the case of slow batch reactor, Pt-oxides and hydroxides mixture can be obtained if the reduction time is insufficient.
5. As it was shown, it is possible to obtain elongated nanoparticles of Pt using TSC. This confirms that the microwaves are able to change the process of PtNPs formation. We suggest that this is directly related to the fact, that the water (as a solvent) is also involved in the process. The increase of temperature changes the properties of water; ergo this opens a novel process window for the nanoparticle shape control.
6. It is well known, that elongated nanoparticles have one face more exposed. Thanks to that such nanoparticles will exhibit different catalytic properties.
7. Elongated nanoparticles after synthesis are tangled. The application of ultrasounds allows forming back stable aqueous suspension. After 6 months, no re-aggregation of these nanoparticles was observed at room temperature. This confirms that the nanoparticles were physically tangled.

## Supplementary Information

Supplementary Information
