# Supplementary material for: The influence of dielectric permittivity of water on the shape of PtNPs synthesized in high-pressure high-temperature microwave reactor
Source: Sci Rep. 2021 Mar 1;11:4851. doi: 10.1038/s41598-021-84388-2 (PMC7921409; doi:10.1038/s41598-021-84388-2)
Supplement: Supplementary file 1 — Supplementary Information [file 41598_2021_84388_MOESM1_ESM.docx]

**Supplementary materials:**

**Title:**

The influence of dielectric permittivity of water on the shape of PtNPs synthesized in high-pressure high-temperature microwave reactor.

**Authors:**

Marek Wojnicki^1,*^, Magdalena Luty-Błocho^1^, Przemysław Kwolek^2^, Marta Gajewska^3^, Robert P. Socha^4^, Zbigniew Pędzich^5^, Edit Csapó^6,7^, Volker Hessel^8^

^1^ AGH University of Science and Technology, Faculty of Non-Ferrous Metals, Mickiewicza Ave. 30, 30-059 Krakow, Poland

^2^ Department of Materials Science, Faculty of Mechanical Engineering and Aeronautics, Rzeszow University of Technology, Aleja Powstańców Warszawy 12,35-959 Rzeszów, Poland

^3^ AGH University of Science and Technology, Academic Centre for Materials and Nanotechnology, al. A. Mickiewicza 30, 30-059 Krakow, Poland

^4^ Institute of Catalysis and Surface Chemistry Polish Academy of Sciences, Niezapominajek 8, 30-239 Krakow, Poland

^5^ AGH University of Science and Technology, Faculty of Materials Science and Ceramics, al. A. Mickiewicza 30, 30-059 Krakow, Poland

^6^ MTA-SZTE Biomimetic Systems Research Group, University of Szeged, H-6720 Dóm tér 8, Szeged, Hungary

^7^ University of Szeged, Department of Physical Chemistry and Materials Science, H-6720, Rerrich B. tér 1, Szeged, Hungary

^8^ The University of Adelaide, School of Chemical Engineering and Advanced Materials, Adelaide, Australia

*Corresponding author, tel. +4812-617-41-26, fax: +4812-633-23-16, e-mail: *[marekw@agh.edu.pl](mailto:marekw@agh.edu.pl)* (Marek Wojnicki)

1. **DLS analysys.**

Zeta potential, as well as size and size distribution of the obtained PtNPs, were determined using the DLS method (Malvern Zetasizer Nano ZS with 630 nm laser). PtNPs size distribution determined using DLS is shown in Fig. 1S.

Fig. 1S. Size distribution by number. Experimental conditions: [Pt(IV)]=1×10^−4^ [TSC]=2×10^−3^ M. Tmax=170^o^C.

For the same sample, Zeta potential measurements were performed. The obtained results are shown in Fig. 2S.


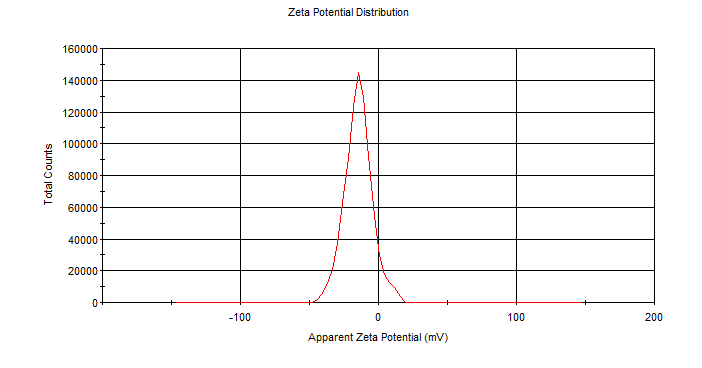


Fig. 2S . Zeta potential distribution. Experimental conditions: [Pt(IV)]=1×10^−4^ [TSC]=2×10^−3^ M. Tmax=170^o^C.

In the case, where elongated nanoparticles were obtained the DLS analysis also shows the two-mode distribution. Due to the specificity of DLS measurement, we present three modes of analysis. The results are shown in Fig. 3S.


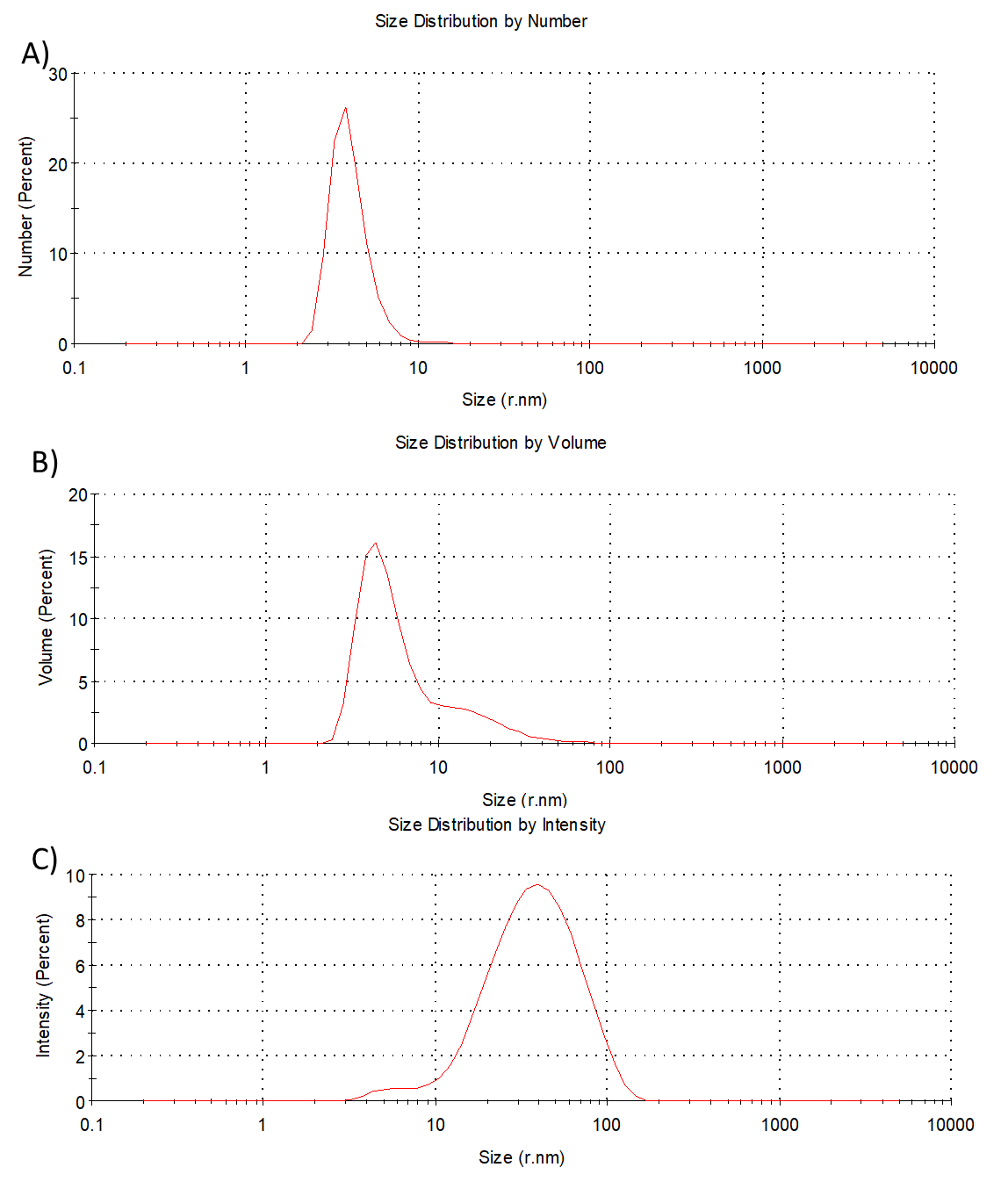


Fig. 3 S. A) nanoparticles size distribution by number, B) by Volume, C) by intensity.

It is well known, that the analysis using these three modes, give slightly different information. In the case of nanowires or particles with elongated shape, two-mode distributions are observed. Where the first peak corresponds to the diameter of e.g. nanowires, while the second one corresponds to the length. Unfortunately, DLS cannot distinguish whether these particles are nanowires or a mixture of small and large particles. Therefore, HR-TEM or HR-SEM measurements are required to verify the observations.

1. **Surface area calculation and comparison with BET**

The number of particles with the radius R can be calculated using equation .

$$N_{i}=\frac{\left(C_{0,Pt(IV)}\cdot V\cdot M_{m,Pt}\right)}{\rho_{Pt}\cdot\frac{4}{3}\cdot\pi R_{i}^{3}}$$

where:

N_i_ – number of particles in R_i_ radius

M_m,Pt_ − molar mass of Pt

C_0,Pt(IV)_ –concentration of platinium(IV)

V – volume of suspension

ρ_Pt_ – platinum density for fcc structure ( 21g/cm^3^)

R_i_ – radius of “i” fraction of Pt nanoparticles

The number of particles as a function of their radius can be used to determine surface area, as a fallows:

$$S=N_{i}\cdot4\cdot\pi\cdot R_{i}^{2}$$

In our case, average nanoparticle radius was assumed as 4.4 nm according to HR-TEM. Calculated value of surface area is equal to 32.32m^2^/g. This vale is in good agreement with BET results which gives 31.99m^2^/g.

1. **HR-TEM analysis.**

Additionally, the EDS analysis were performed. The aim of this studies is to confirm, that there is no impurities in studies material. Obtained result are shown in Fig. 3S and Fig. 4S.





Fig. 4 HR-TEM analysis of the sample.

For the selected area the EDS analysis was performed. The obtained results are shown below (see Fig. 4S).


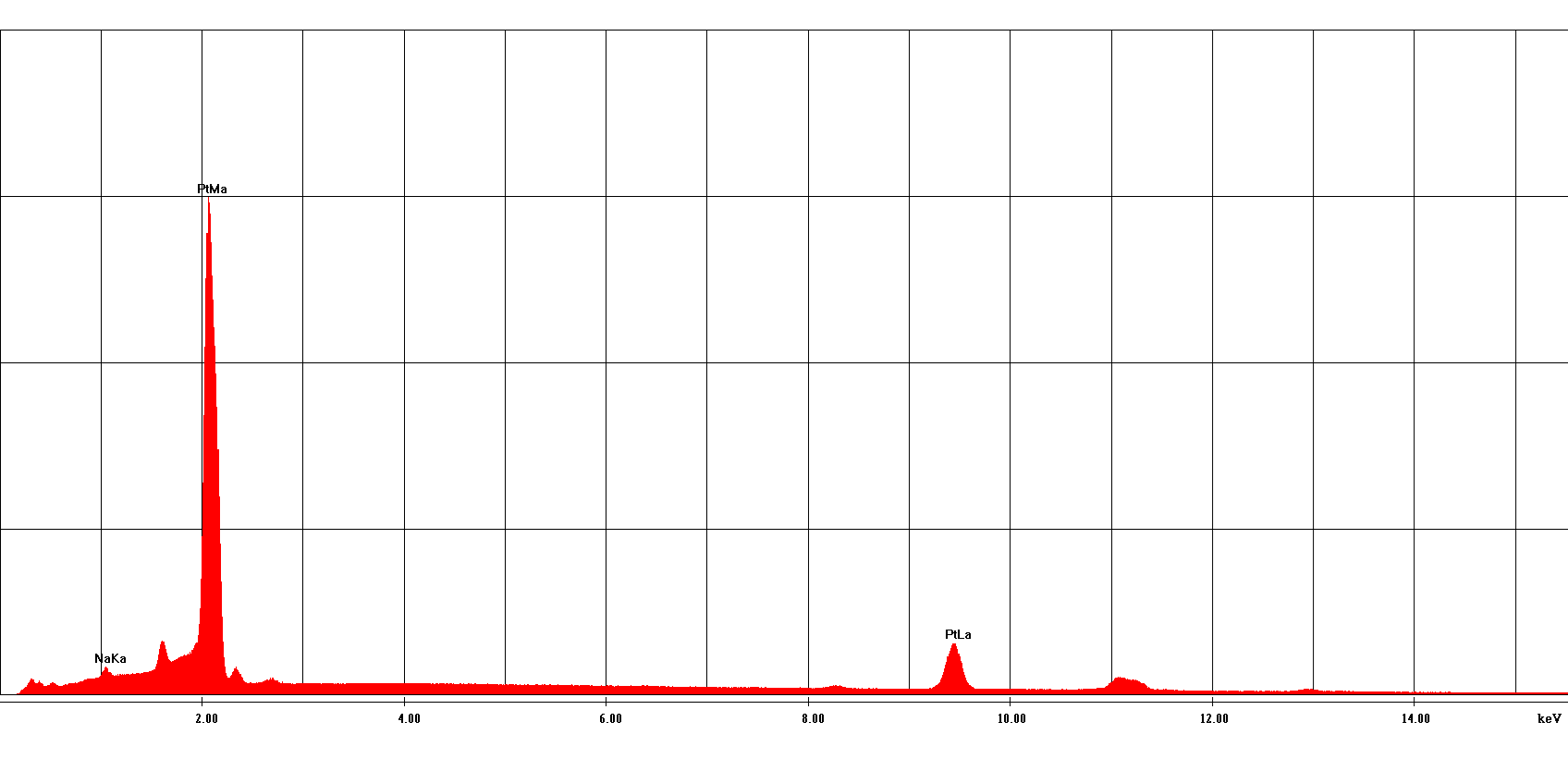


Fig. 5 EDS analysis of the sample.

As it can be seen, the only peak from Pt and Na are observed (pikes from C, O were omitted).
